# Comparative Investigation of Composition, Antifungal, and Anti-Inflammatory Effects of the Essential Oil from Three Industrial Hemp Varieties from Italian Cultivation

**DOI:** 10.3390/antibiotics10030334

**Published:** 2021-03-22

**Authors:** Giustino Orlando, Sabrina Adorisio, Domenico Delfino, Annalisa Chiavaroli, Luigi Brunetti, Lucia Recinella, Sheila Leone, Marianna D’Antonio, Gokhan Zengin, Alessandra Acquaviva, Mirko Antico, Paola Angelini, Giancarlo Angeles Flores, Roberto Venanzoni, Massimo Tacchini, Simonetta Cristina Di Simone, Luigi Menghini, Claudio Ferrante

**Affiliations:** 1 Department of Pharmacy, Botanic Garden “Giardino dei Semplici”, Università degli Studi “Gabriele d’Annunzio”, via dei Vestini 31, 66100 Chieti, Italy; giustino.orlando@unich.it (G.O.); annalisa.chiavaroli@unich.it (A.C.); luigi.brunetti@unich.it (L.B.); lucia.recinella@unich.it (L.R.); sheila.leone@unich.it (S.L.); alessandra.acquaviva@studenti.unich.it (A.A.); mirko.antico@studenti.unich.it (M.A.); disimonesimonetta@gmail.com (S.C.D.S.); claudio.ferrante@unich.it (C.F.); 2 Section of Pharmacology, Department of Internal Medicine, Università degli Studi di Perugia, 06100 Perugia, Italy; adorisiosabrina@libero.it (S.A.); domenico.delfino@unipg.it (D.D.); 3 Bioinvest S.r.l., via Filippo Masci, Building 6, 66100 Chieti, Italy; dantoniomarianna2@gmail.com; 4 Department of Biology, Science Faculty, Selcuk University, Campus, 42130 Konya, Turkey; gokhanzengin@selcuk.edu.tr; 5 Veridia Italia Srl, Via Raiale 285, 65100 Pescara, Italy; 6 Department of Chemistry, Biology and Biotechnology, University of Perugia, 06100 Perugia, Italy; paola.angelini@unipg.it (P.A.); giancarlo.angelesflores@studenti.unipg.it (G.A.F.); roberto.venanzoni@unipg.it (R.V.); 7 Department of Life Sciences and Biotechnology (SVeB), UR7 Terra&Acqua Tech, University of Ferrara, 44121 Ferrara, Italy

**Keywords:** industrial hemp, essential oil, inflammation, dermatophytes, gene expression, bioinformatics

## Abstract

Industrial hemp is characterized by a huge amount of by-products, such as inflorescences, that may represent high-quality sources of biomolecules with pharmaceutical interest. In the present study, we have evaluated the phytochemical profile, including terpene and terpenophenolic compounds, of the essential oils (EOs) of *Futura 75*, *Carmagnola selezionata* and *Eletta campana* hemp varieties. The EOs were also tested for antifungal properties toward *Trichophyton mentagrophytes, Trichophyton rubrum, Arthroderma crocatum, Arthroderma quadrifidum, Arthroderma gypseum, Arthroderma curreyi,* and *Arthroderma insingulare*. In parallel, we investigated the inhibitory effects of the EOs against tyrosinase, and the production of prostaglandin E_2_ in isolated mouse skin exposed to hydrogen peroxide. In human H1299 lung adenocarcinoma cells, we also evaluated the influence of the EOs on the gene expression of angiotensin-converting enzyme 2 (ACE2) and transmembrane protease serine 2 (TMPRSS2), which are involved in SARS-CoV-2 entry in human host. *E*-caryophyllene and α-pinene were the prominent terpenes in the EOs, whereas the cannabidiolic acid was the terpenophenol present at higher concentration. The EOs inhibited the growth of all tested dermatophytes species. In isolated skin specimens, EOs prevented the hydrogen-peroxide-induced synthesis of prostaglandin E_2_, consistent with the intrinsic antityrosinase activity. Finally, in H1299 cells, all tested EOs reduced the gene expression of ACE-2 and TMPRSS2, as well. Therefore, the present findings highlight the rationale for the use of the present EOs against infectious diseases.

## 1. Introduction

Industrial hemp (*Cannabis sativa* L.) is a traditional multiuse crop that has been extensively cultivated throughout the history as a valuable source of fibers and nutrients, rather than for the extraction of the Δ^9^-tetrahydrocannabinol (THC) [1,2]. THC represents the sole recognized psychotropic phytocompound present in the industrial hemp cultivars, which are bred to produce THC in traces (≤0.3 % *w/w*). However, recent findings suggest the presence of other terpenophenolics potentially endowed with THC-like effects [3]. Currently, only hemp varieties that are certified for the THC content <0.2% are permitted to be cultivated in the European Union (EU Regulation N°1124/2008-12 November 2008; Italian Regulation N°172/2017). The Italian Government also promoted the cultivation of industrial hemp under an agricultural policy aimed to implement environmentally friendly crops and contrast the loss of agricultural lands. The application of this strategy is particularly evident in the middle Italy regions, including Abruzzo, where industrial hemp is the object of a renewed interest, as indicated by the increased surface dedicated to the cultivation. The national and supranational interests toward this crop have led to the development of a huge number of new varieties that, besides being certified for the low THC content, are sources of many hemp-seed-deriving foods, characterized by the valuable content in vitamins, minerals, proteins, carbohydrates, and lipids. In this regard, it is sensitive to highlight the seed content in fatty acids, namely linoleic (ω-6) and linolenic (ω-3), often in the 3:1 ratio, which is ideal for the prevention of chronic inflammatory diseases, in humans [4]. However, the industrial hemp chain is also characterized by a huge number of by-products, such as inflorescences, which may represent high-quality sources for extracting biomolecules of pharmaceutical interest, thus potentially contributing to the improvement of the whole botanical chain production [5]. Although the inflorescences have long been considered as waste material to be sold in the dried form for the sole technical use [6], their inclusion as tonics in beverages or flavorings and nutraceuticals in certified foods has been suggested [7]. The valorization of hemp inflorescences is also supported by recent studies, pointing out the use of inflorescences as sources of bioactive extracts and biomolecules, including terpenes, terpenophenolics, and phenolic compounds [8,9,10,11,12,13]. In this regard, inflorescence essential oils (EOs) have been described as effective insecticide, antibacterial, and antiproliferative agents [8,14], whereas the water extracts displayed anti-inflammatory and antityrosinase properties together with intriguing inhibitory effects on the growth of multiple fungi strains, including *Candida* and dermatophytes species [9,10,11]. Very recently, industrial hemp extracts characterized by different polarities have also been explored as anti-COVID-19 agents, with particular regards to the inhibitory effects on the gene expression of angiotensin-converting enzyme 2 (ACE2) and transmembrane protease serine 2 (TMPRSS2) [12,13], which are reputed to play master roles in mediating SARS-CoV-2 virus entry in the human host [15,16]. Aiming to enlarge our comprehension about antimicrobial properties of industrial hemp by-products, in the present multidirectional study, we have tested the antimycotic properties of three industrial hemp varieties, namely, *Futura 75, Carmagnola selezionata*, and *Eletta campana,* toward several dermatophytes species (*Trichophyton mentagrophytes*, *Trichophyton rubrum*, *Arthroderma crocatum, Arthroderma quadrifidum*, *Arthroderma gypseum*, *Arthroderma curreyi*, and *Arthroderma insingulare*) which are reputed to be involved in skin inflammatory disorders, including hyperpigmentation [17,18,19]. In parallel, we have evaluated the inhibitory effects of the EOs against the activity of tyrosinase, an enzyme playing a master role in hyperpigmentation [20], and the production of prostaglandin E_2_ in an *ex vivo* toxicity model constituted by isolated mouse skin specimens challenged with hydrogen peroxide [21]. Also considering the recent interest of scientific research toward hemp-deriving extracts as putative anti-COVID-19 agents [12,13], we investigated the influence of the aforementioned EOs on the gene expression of ACE-2 and TMPRSS2 in human H1299 lung adenocarcinoma cell line. In parallel, we studied the effects of the EOs on cell viability and the gene expression of apoptosis regulators, namely transforming growth factor (TGF)-β and B-cell lymphoma 2 (Bcl-2) [22,23], which could be targeted by hemp phytocompounds [24]. Finally, detailed phytochemical analyses were conducted for unravelling the composition of the EOs in terms of terpene and terpenophenolic compounds.

## 2. Results and Discussion

**Figure 1:**
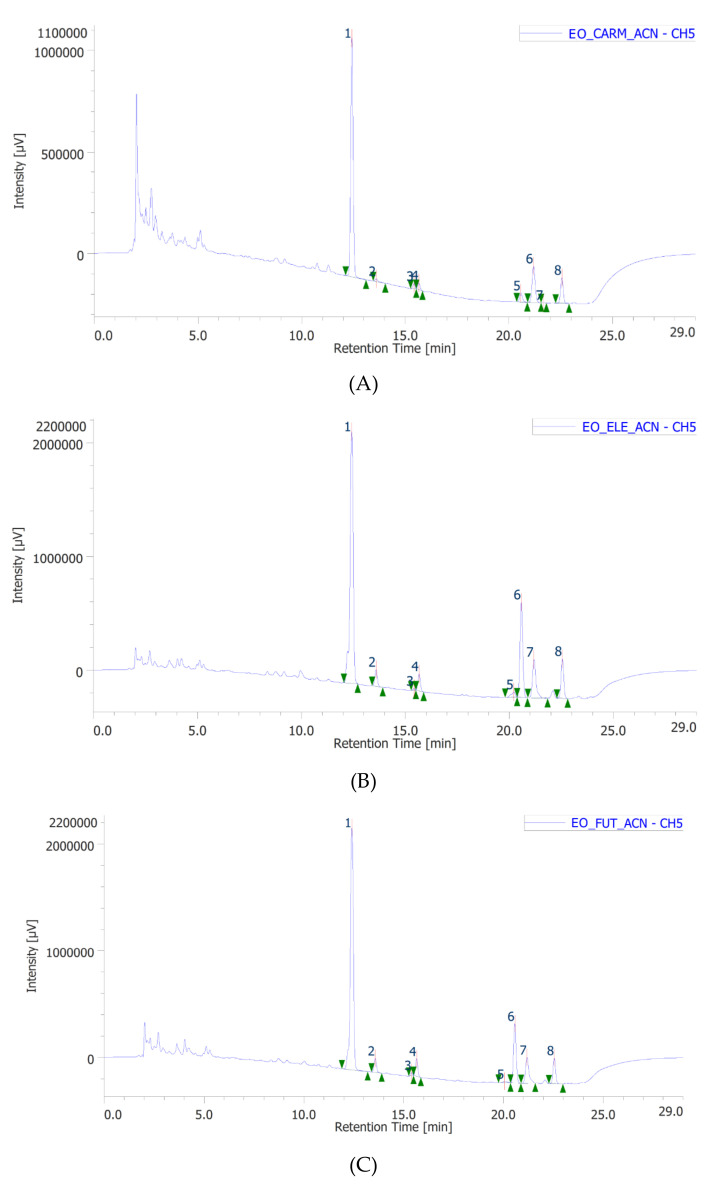
High-performance liquid chromatography coupled to ultraviolet and mass spectrometry detectors (HPLC–UV-MS) analysis of *Carmagnola selezionata* (**A**)*, Eletta campana,* (**B**) and *Futura 75* (**C**) terpenophenols. In all tested essential oils, cannabidiolic acid (1) was the prominent phytocompound compared to the other identified terpenophenols. Specifically, the cannabidiolic acid was present in the range 57.4–70.6%, corresponding to 24.6–62.9 µg/mg EO.

In the present study, a multidirectional approach was followed for exploring phytochemical composition and pharmacological applications of the EOs of the industrial hemp varieties *Futura 75, Eletta campana,* and *Carmagnola selezionata*. Recently, we characterized the terpene profile of the aforementioned EOs [25], and the chromatographic analyses indicated the *E*-caryophyllene and α-pinene as the most representative terpenes, in all tested EOs. These results agreed with previous literature data [8,26], as well. The gas chromatography coupled to mass spectrometry detector (GC–MS) profile of the three EOs is also available in Table 1, Table 2 and Table 3, whereas the high-performance liquid chromatography coupled to ultraviolet and mass spectrometry detectors (HPLC–UV-MS) chromatograms show the cannabidiolic acid as the prominent terpenophenol (range 57.4–70.6%), in all tested samples (Figure 1A–C). This is consistent, albeit partially, with literature data highlighting the presence of terpenophenols in the EO of industrial hemp [27]. However, the study conducted by Nagy et al. [27] showed the cannabidiol as the main terpenophenol in the EO (24.9%) of the *Vavilov* variety, whereas in the present cultivars, the cannabidiol was present at much lower percentages (1.79–3.58%). By contrast, in the *Vavilov* variety, cannabidiolic acid was identified in the ethyl acetate extract but not in the EO. We cannot exclude that these discrepancies could depend upon either variability in the plant’s secondary metabolism or harvesting/extractive conditions. The diverse pattern in cannabinoid content is also mirrored by differences in the *E*-caryophyllene yield that was higher (28.9%) in the EO of *Vavilov* variety, compared to the present EOs (13.5–19.3%). Given our recent findings about the pharmacological properties of the industrial hemp (*Futura 75* variety) water extract as antioxidant/anti-inflammatory, antityrosinase, and antidermatophytes agent against *T. rubrum*, *T. interdigitale,* and *Microsporum gypseum* [10,11], in the present study, a deeper investigation about the antimycotic properties of the EOs was conducted on a broader spectrum of pathogen strains, namely *T. mentagrophytes*, *Trichophyton rubrum*, *Arthroderma crocatum*, *Arthroderma quadrifidum*, *Arthroderma gypseum*, *Arthroderma curreyi*, and *Arthroderma insingulare*. As depicted in Table 4, the EOs were effective in inhibiting the growth of all tested dermatophytes species, with IC_50_ values in the range 0.312–10 µg/mL. Overall, *Futura 75* variety displayed the higher mycostatic activity, compared to the other two cultivars, although all EOs were generally more potent than the reference antimycotic drug griseofulvin. The mycostatic effects induced by the EOs also agree with the efficacy of caryophyllene oxide as antimycotic, in an in vitro model of onichomycosis [28]. 

**Figure 2:**
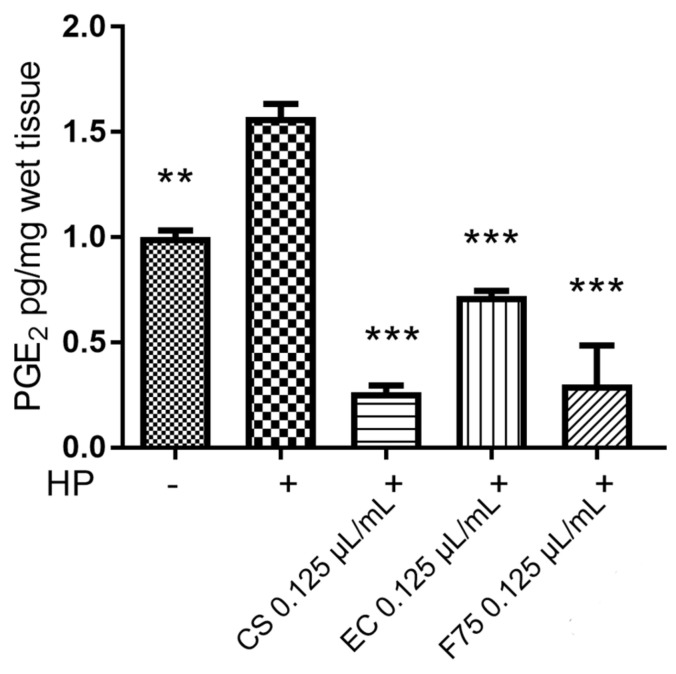
Inhibitory effect induced by the essential oils (EOs: 0.125 µL/mL) of *Carmagnola selezionata* (CS), *Eletta campana* (EC), and *Futura 75* (F75) cultivars on hydrogen-peroxide (HP)-induced prostaglandin E2 (PGE_2_) level (pg/mg wet tissue), in isolated mouse skin specimens. ANOVA, *p* < 0.0001; ** *p* < 0.01, *** *p* < 0.001 vs. hydrogen peroxide (HP) group.

In view of potential applications as skin protective agents, the EOs were assayed for measuring their intrinsic antityrosinase activity. Tyrosinase is a key enzyme involved in the melanogenesis process; therefore, the enzyme inhibition could be crucial to counteract inflammatory-induced skin hyperpigmentation, especially during aging [28]. *Eletta campana* was the most active as enzyme inhibitory agent (Table 5), and this could be related, albeit partially, to its higher scavenging/reducing activity (Table 6) [25]. Plant secondary metabolites have been reported to exert antioxidant and enzyme inhibitory effects, and high correlation also exists between radical scavenging and enzyme inhibition [29]. Overall, the antidermatophytes and antityrosinase effects induced by hemp EOs suggest protective effects against skin inflammatory disorders that have been partly explored through an ex vivo approach. In this regard, isolated mouse skin specimens were exposed to hydrogen peroxide 1 mM and treated with 0.125 µL/mL (about 100 µg/mL) of EOs. In isolated skin tissue, the hydrogen peroxide stimulus is able to induce both tyrosinase and prostaglandin E_2_ (PGE_2_) synthesis [21]. In this context, the blunting effect induced by all tested EOs on hydrogen-peroxide-induced PGE_2_ level (Figure 2) further supports the use of hemp EOs against infectious and inflammatory skin diseases.

**Figure 3:**
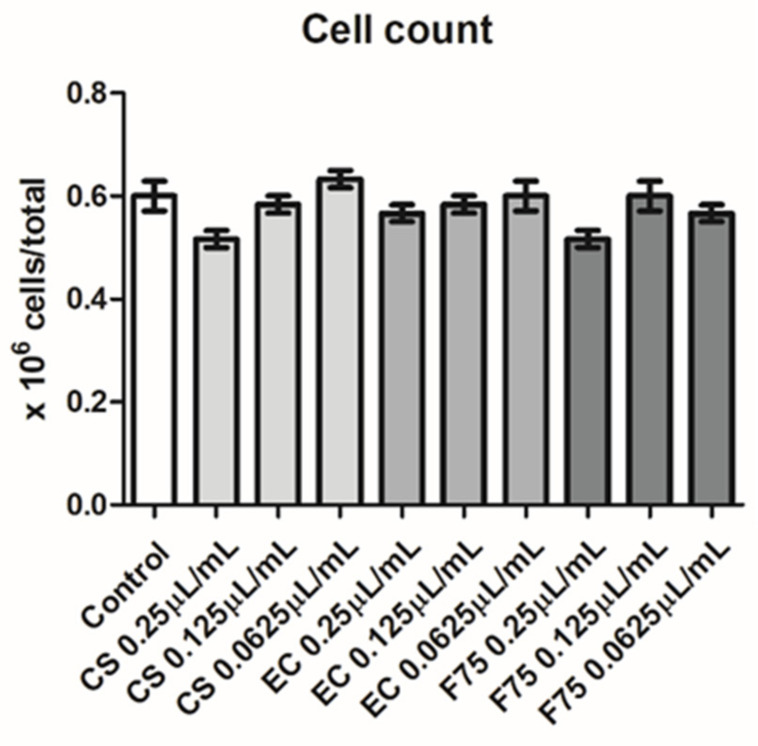
Null effects of the industrial hemp essential oils (CS), (EC), and (F75) (0.0625–0.25 µL/mL) on the viability of the human H1299 lung adenocarcinoma cell line.

**Figure 4:**
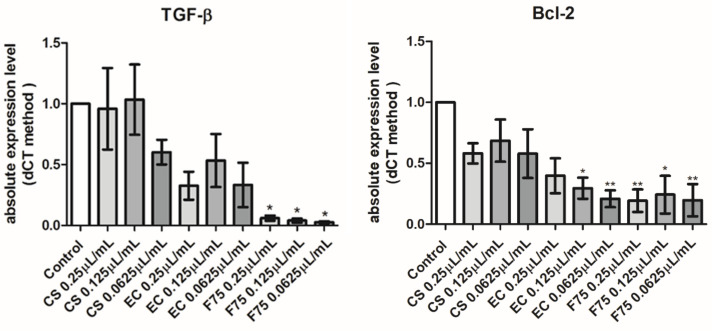
Real-time PCR analyses from three independent experiments (mean ± SEM) of Bcl-2 and TGF-β, in cells treated with vehicle (Control) or 0.25, 0.125, and 0.0625 μL/mL of essential oils (CS), (EC), and (F75). Differences between groups were evaluated using one-way ANOVA test. Differences were considered statistically significant according to the following criteria: * *p* < 0.05; ** *p* < 0.01.

**Figure 5:**
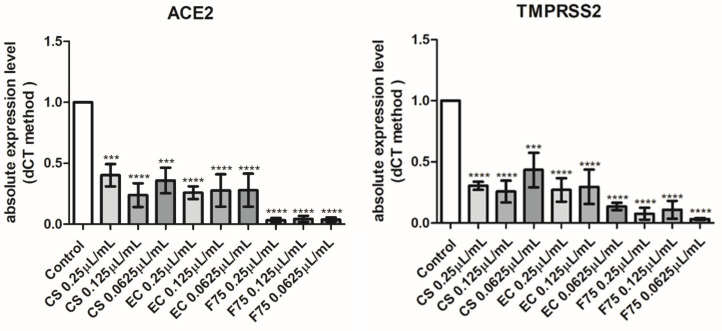
Real-time PCR analyses from five independent experiments (mean ± SEM) of ACE2 and TMPRSS2, in cells treated with vehicle (Control) or 0.25, 0.125, 0.0625 μL/mL of essential oils (CS), (EC), and (F75). Differences between groups were evaluated using one-way ANOVA test. Differences were considered statistically significant according to the following criteria: *** *p* < 0.001, **** *p* < 0.0001.

**Figure 6:**
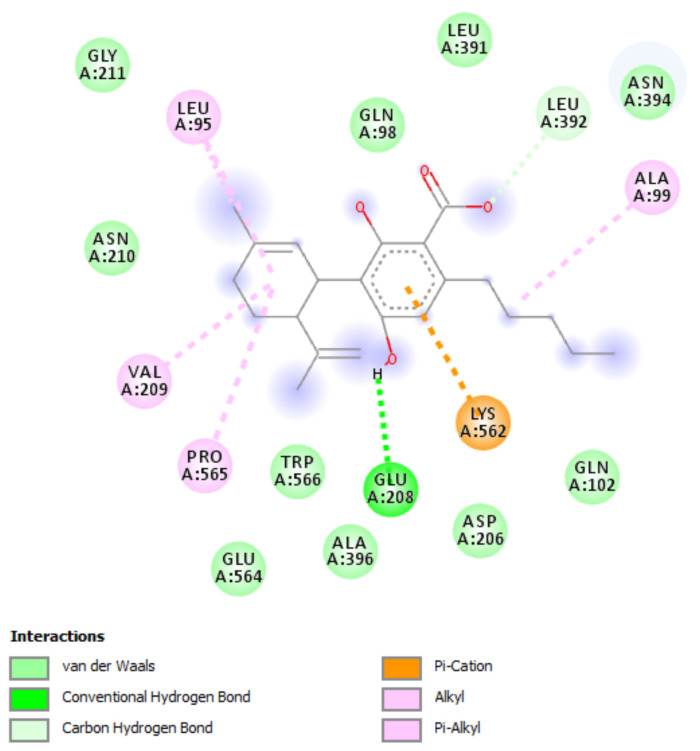
Putative interactions between cannabidiolic acid and angiotensin-converting enzyme 2 (ACE2; PDBID: 1R4L). Free energy of binding (ΔG) and affinity (Ki) are −7.3 kcal/mol and 4.5 µM, respectively.

Hemp EOs were also investigated in an in vitro model constituted by human H1299 lung adenocarcinoma cells. As depicted in Figure 3, the EOs (0.0625–0.25 µL/mL) did not alter cell number following pharmacological treatment, thus excluding any significant effect on cell survival, in basal conditions. On the other hand, the literature’s data suggest antiproliferative effects exerted by hemp EOs tested in a similar concentration range [8]. However, the capability of the EO to reduce the cell viability under the limit of biocompatibility (70% viability) could be related to the tumor-cell-line sensitivity [8]. In the present study, the null effect on cell viability is also mirrored by the EOs-induced simultaneous inhibition of the gene expression of both Bcl-2 and TGFβ (Figure 4), which are known to regulate apoptosis in opposite ways [30,31,32]. Regarding the *Futura 75* variety, the inhibition of Bcl-2 and TGFβ was statistically significant at all tested concentrations, whereas the EOs of the other two cultivars did not significantly alter Bcl-2 and TGFβ gene expression. Recent studies explored the potential of industrial hemp polar extracts as anti-COVID-19 agents, with particular regards to the gene expression inhibition of ACE2 and TMPRSS2 [12,13], which, besides being co-expressed [33], are reputed to play master roles in mediating SARS-CoV-2 virus entry in the human host [15,16]. This is as also summarized by the pathway map hsa05171 deposited on the KEGG PATHWAY Database (https://www.genome.jp/kegg/pathway.html). Considering the expression of both ACE2 and TMPRSS2 in H1299 cells [22], hemp EOs were assayed for evaluating their influence on ACE2 and TMPRSS2 gene expression, as well. As showed in Figure 5, all investigated EOs were able to significantly inhibit the gene expression of ACE2 and TMPRSS2 in H1299 cells exposed to the treatment, in basal conditions. Considering the results of the quantitative phytochemical analysis, a docking approach was also conducted on the sole ACE2 (PDBID: 1R4L), with the aim of exploring putative direct interactions between ACE2 and the main EO terpenophenol, namely cannabidiolic acid. Indeed, the molecular structure of TMPRSS2 is currently unknown, and the bioinformatics data and predictions available in literature [34] and database (STRINGH, SWISSMODEL) are based on the use of hepsin (PDB ID: 1Z8G), which is currently chosen for its homology with TMPRSS2. The result of the present docking runs yielded putative affinity (Ki) in the micromolar range (Figure 6), thus suggesting binding interactions of cannabidiolic acid with ACE2, which could partly explain the pattern of gene expression observed following treatment. Overall, these findings suggest the hemp EOs as promising agents to be further investigated with the final goal of optimizing their use in protective devices for counteracting the SARS-CoV-2 virus entry in the human host.

## 3. Materials and Methods

### 3.1. Pharmacognostic Analysis

Three selected cultivars of industrial hemp, namely *Cannabis sativa* L. cultivar “*Futura 75*” (F75), *C. sativa* L. cultivar “*Eletta campana*”, and *C. sativa* L. cultivar “*Carmagnola selezionata*” were cultivated in experimental crops, in Abruzzo region, and more specifically in the surrounding of the Pescara river valley, Italy (Table 7). This valley is a typical piedmont-hilly zone of the central-eastern Apennines, which originates from the ecotone between the Gran Sasso and Maiella-Morrone massifs, in the northern and southern part, respectively, and declines within 45 km in an extended drainage basin to the Adriatic coast. The basin area expresses the Mediterranean bi-climate characters typical of the Adriatic coast with dry summer characterized by law rainfall. The total extension of the experimental crops were 24,000 square meters on level ground, with a mean slope lower than 5% and altitude range 70–100 m asl. The planting scheme was in lines with a central 2 m irrigation strip widths.

In order to improve the development of plants according to the high temperature and long daylight, the sowings were performed between mid and late March 2019, with a prevision to reach a scalar blooming stage, ready for harvesting, in late August/September. The cultivar *Eletta campana* is a dioecious Italian selection deriving from original crops of the early 1900 s, largely cultivated in the south of Italy for fiber production. It is in the European community list of varieties admitted for cultivation and its agronomic needs fit with the Mediterranean environment. It is considered to be a late-flowering cultivar that produces a high amount of biomass, due to the large leaf surface and compact smelling inflorescences, endowed with a rich presence of terpenes. The cultivar name refers to Campania region, and the selected cultivar is largely cultivated throughout central and south Italy. Similarly, *Carmagnola selezionata* is a dioecious cultivar originally selected for the production of fiber. The mature plants can grow over 6 meters but are also adapted for seeds and flowers production. *Futura 75* is a French selection of monoecious variety for seeds and biomass production, characterized by high adaptability for multipurpose cultivation. Previous experiences of cultivation confirmed the suitability of this cultivar for local environmental conditions, also in central Italy [8]. All selected seeds are present in the plant variety database of the European Commission in the agricultural species list. The genus *Cannabis* and specifically the selected cultivars are characterized by high adaptability, and based on previous experiences, no specific agronomical practices were planned but just a superficial soil preparation (ploughing, disc harrowing, and milling), automated sowing for optimized seed density, no fertilization, and only three programmed automated irrigation cycles. During the phase of flowering, an accurate manual collection of only mature flowers was done. The collection was repeated daily to obtain only high-quality mature flowers. Fresh plant material was immediately transferred to the laboratory and extracted within 6 hours. Random samples from collections were selected and botanical identity was confirmed by classical taxonomical approach (done by Prof. L. Menghini, Full Professor in Pharmaceutical Botany at the Department of Pharmacy, “G. d’Annunzio” University, Chieti, Italy). Fresh inflorescences were distributed to obtain a homogeneous dispersion in the distillation chamber in a stainless-steel distillatory Clevenger-type apparatus (Albrigi Luigi S.r.l., Stallavena, Italy). The volumetric EO yields (%, *v/w*) were determined and expressed for phytochemical (mL in plant fresh weight) or agronomical (mL from hectare) interpretations. Samples of EOs to be used for analytical characterization and biological tests were dehydrated through a passage in anhydrous sodium sulfate and stored in a dark glass bottles with a PTFE septa cap at 4 °C until used. 

### 3.2. HPLC–UV-MS Determination of Terpenophenolic Compounds

Industrial hemp EOs were analyzed for terpenophenol quantitative determination using a reversed-phase HPLC–UV-MS in gradient elution mode. HPLC: the separation was conducted within 30 minutes, starting from the following conditions: 0.007% formic acid, 7% water, and 93% acetonitrile. The details about gradient are listed in Table 8. The separation was performed on an Infinity lab Poroshell 120 reverse phase column (C18, 150 mm × 4.6 mm i.d., 2.7 µm) (Agilent Santa Clara, CA, USA). Column temperature was set at 30 °C. The EOs were qualitatively analyzed with MS detector in positive ion mode. MS signal identification was realized through comparison with standard solutions and MS spectra present in the MassBank Europe database (https://massbank.eu/MassBank/). Specifically, the mass-to-charge ratio (*m/z*), the wavelength, and retention times considered for compound identification are listed in Table 9.

### 3.3. Antityrosinase Activity

The enzyme inhibition toward tyrosinase was conducted with colorimetric assay, as well, and expressed as kojic acid equivalents. The experimental procedures for all these assays were comprehensively described in our previous study [35].

### 3.4. Antimycotic Activities

The industrial hemp EOs were assayed for antimycotic effects against different dermatophytes species: *Trichophyton mentagrophytes* (CCF 4823), *Trichophyton rubrum* (CCF 4879), *Trichophyton rubrum* (CCF 4933), *Arthroderma crocatum* (CCF 5300), *Arthroderma quadrifidum* (CCF 5792), *Arthroderma gypseum* (CCF 6261), *Arthroderma curreyi* (CCF 5207), and *Arthroderma insingulare* (CCF 5417). The detailed protocol of the present antimycotic evaluations is fully described in recent published papers of ours [36]. The antimycotic effects were compared to the reference drug griseofulvin.

### 3.5. Ex Vivo Model of Hydrogen-Peroxide-Induced Toxicity in Isolated Mouse Skin Tissue

Twenty male adult C57BL6 mice (20–25 g) were sacrificed by CO_2_ inhalation (100% CO_2_ at a flow rate of 20% of the chamber volume per min), and skin specimens were immediately collected from the back of the animals and maintained in a humidified incubator with 5% CO_2_ at 37 °C for 4 h, in DMEM buffer with the addition of hydrogen peroxide (1 mM). The experimental procedure was approved by the Italian Ministry of Health (Authorization Number: F4738.N.5QP). During the incubation period, skin specimens were stimulated with different industrial hemp EOs (0.125 μL/mL). Tissue supernatants were collected, and the PGE_2_ level (ng/mg wet tissue) was measured by radioimmunoassay (RIA), as previously reported [37].

### 3.6. Cell Culture

The human H1299 lung adenocarcinoma cell line was cultured with RMPI-16140 medium supplemented with 10% heat-inactivated fetal bovine serum, 100 U/mL penicillin, and 100 μg/mL streptomycin. Cells were incubated at 37 °C with 5% CO_2_. The H1299 cell line was purchased from ATCC (Manassas, VA, United States). In the experiments, cells were seeded into 6 well culture plates, kept ad concentration of 2 × 10^5^ cells/mL, and after 24 h, were treated with different concentrations of essential oils for 24 h at the final concentrations reported in the figures.

### 3.7. Real-Time Reverse Transcription Polymerase Chain Reaction (Real-Time RT PCR)

Total RNA was extracted from cells by RNeasy Plus Micro Kit (Qiagen), and generation of cDNA was performed in triplicate using a QuantiTect reverse transcription kit (Qiagen). All reactions were performed using an ABI-7300 real-time Cycler, and amplification was performed using TaqMan Assay (Hs01085333 for ACE2, Hs01122322 for TMPRSS, HS 00608023 for Bcl-2, HS 00998133 for TGFβ, and eukaryotic 18S rRNA as an endogenous control). 

### 3.8. Bioinformatics

Docking calculations were conducted through the Autodock Vina of PyRx 0.8 software, as recently described [38,39]. Crystal structures of target proteins were derived from the Protein Data Bank (PDB) with PDB ID as follows: 1R4L (inhibitor-bound human angiotensin-converting enzyme-related carboxypeptidase (ACE2)). Discovery studio 2020 visualizer was employed to investigate the protein–ligand nonbonding interactions.

### 3.9. Statistical Analysis

The statistical significance of difference between controls and experimental groups was evaluated using one-way analysis of variance (ANOVA). Results were expressed as means ± SD (standard deviation). Statistical analysis was performed using GraphPad Prism™ (Version 6.01) software (GraphPad Software, San Diego, CA, USA). A *p*-value < 0.05 was considered as statistically significant.

## 4. Conclusions

In conclusion, the present findings highlight the rationale for improving the whole botanical chain of the industrial hemp, considering the inflorescence by-product as a valuable source of biomolecules with pharmacological applications. Specifically, the present study aims to be a starting point for the development of innovative topical pharmaceutical preparations and devices. Indeed, the industrial hemp EOs could be included in dermatological formulations for counteracting the inflammatory burden of oxidative stress and inflammation occurring during dermatophytes infections. Additionally, considering the inhibitory effects on ACE2 and TMPRSS2 gene expression, we hypothesize the inclusion of the present EOs in protection devices, such as chirurgical masks, functioning as physical barriers against COVID-19.

## Figures and Tables

**Table 1:** Gas chromatography coupled to mass spectrometry detector (GC–MS) analysis of *Eletta campana* EO [25].

Compound	Area %	RI ^a^	RIL ^b^
α-pinene	11.9	934	933
camphene	0.3	949	953
β-pinene	3.6	977	978
myrcene	6.8	994	991
α-phellandrene	0.1	1006	1007
δ-3-carene	0.5	1011	1009
α-terpinene	0.1	1019	1018
para-cymene	0.2	1028	1024
limonene	2.2	1031	1030
eucalyptol	0.4	1040	1032
Z-β-ocimene	0.5	1043	1035
E-β-ocimene	1.9	1053	1046
γ-terpinene	0.2	1062	1058
terpinolene	2.3	1091	1086
linalyl anthranilate	0.2	1110	1104
fenchyl alcohol	0.1	1123	1123
terpinen-4-ol	0.2	1187	1184
α-ylangene	0.2	1378	1371
Z-caryophyllene	0.3	1414	1413
E-caryophyllene	13.5	1428	1424
α-trans-bergamotene	0.6	1442	1432
α-humulene	5.3	1462	1454
9-epi-E-caryophyllene	0.7	1470	1464
γ-muurolene	0.5	1485	1478
α-amorphene	0.2	1488	1482
β-selinene	1.5	1495	1492
α-selinene	1.7	1504	1501
Z-γ-bisabolene	1.4	1516	1511
γ-cadinene	0.4	1523	1512
selina-4(15),7(11)-diene	1.3	1545	1540
selina-3,7(11)-diene	2.5	1552	1546
B-germacrene	0.3	1569	1557
caryophyllene oxide	2.2	1599	1587
humulene epoxide	0.7	1626	1613
α-bisabolol	0.5	1697	1688
tetracosane	6.0	2406	2400
heptacosane	23.9	2690	2700
12 unknown compounds	4.8		

^a^ calculated retention index. ^b^ retention index reported in literature or commercial database.

**Table 2:** GC–MS analysis of *Futura 75* EO [25].

Compound	Area %	RI ^a^	RIL ^b^
α-pinene	14.9	934	933
camphene	0.3	949	953
β-pinene	3.8	977	978
myrcene	11.8	994	991
α-phellandrene	0.2	1006	1007
δ-3-carene	0.5	1011	1009
α-terpinene	0.2	1018	1018
para-cymene	0.1	1028	1025
limonene	1.8	1031	1030
eucalyptol	0.2	1041	1032
E-β-ocimene	2.9	1053	1046
γ-terpinene	0.2	1062	1058
terpinolene	5.1	1091	1086
Z-caryophyllene	0.5	1414	1413
α-cis-bergamotene	0.3	1422	1416
E-caryophyllene	19.3	1428	1424
α-trans-bergamotene	1.9	1442	1432
α-humulene	8.3	1462	1454
9-epi-caryophyllene	1.1	1470	1464
β-selinene	1.7	1495	1492
α-selinene	1.3	1504	1501
selina-4(15),7(11)-diene	0.9	1545	1540
selina-3,7(11)-diene	1.5	1552	1546
caryophyllene oxide	4.3	1599	1587
humulene epoxide	1.1	1626	1613
allo-aromadendrene epoxide	0.4	1650	1644
tetracosane	8.8	2407	2400
13 unknown compounds	6.6		

^a^ calculated retention index. ^b^ retention index reported in literature or commercial database.

**Table 3:** GC–MS analysis of *Carmagnola selezionata* EO [25].

Compound	Area %	RI ^a^	RIL ^b^
α-pinene	12.6	934	933
camphene	0.3	949	953
β-pinene	4.1	977	978
myrcene	26.4	995	991
α-phellandrene	0.3	1006	1007
δ-3-carene	0.3	1012	1009
α-terpinene	0.3	1019	1018
para-cymene	0.2	1028	1025
limonene	4.7	1031	1030
eucalyptol	0.5	1041	1032
Z-β-ocimene	0.4	1043	1035
E-β-ocimene	2.5	1053	1046
γ-terpinene	0.3	1062	1058
terpinolene	7.0	1091	1086
linalyl anthranilate	0.3	1110	1104
fenchyl-alcohol	0.2	1123	1123
Z-caryophyllene	0.4	1414	1413
E-caryophyllene	19.1	1428	1424
α-trans-bergamotene	0.2	1442	1432
α-humulene	7.2	1462	1454
9-epi-E-caryophyllene	0.6	1470	1464
β-selinene	1.3	1495	1492
α-selinene	1.1	1504	1501
Z-γ-bisabolene	1.1	1514	1511
selina-4(15),7(11)-diene	0.3	1545	1540
selina-3,7(11)-diene	0.5	1552	1546
caryophyllene oxide	3.2	1599	1587
humulene epoxide	1.0	1626	1613
10 unknown compounds	3.6		

^a^ calculated retention index. ^b^ retention index reported in literature or commercial database.

**Table 4:** Minimum inhibitory concentration (MIC) of industrial hemp essential oils toward selected dermatophyte strains.

Dermatophytes Species	MIC * (µg/mL)			
	*Carmagnola Selezionata*	*Eletta Campana*	*Futura 75*	*Griseofulvin*
*T. mentagrophytes* (CCF 4823)	0.79(0.625–1.25)	< 0.312	0.79(0.625–1.25)	2.52 (2–4)
*T. rubrum* (CCF 4879)	3.15(2.5–5)	6.3(5–10)	6.3(5–10)	3.175(2–4)
*T. rubrum* (CCF 4933)	0.99(0.625–1.25)	1.57(1.25–2.5)	0.99(0.625–1.25)	1.26(1–2)
*A. crocatum* (CCF 5300)	1.57(1.25–2.5)	1.57(1.25–2.5)	0.39(0.625–0.312)	>8
*A. quadrifidum* (CCF 5792)	3.15(2.5–5)	3.15(2.5–5)	0.39(0.625–0.312)	>8
*A. gypseum* (CCF 6261)	1.57(1.25–2.5)	3.15(2.5–5)	0.49(0.312–0.625)	3.174 (2–4)
*A. curreyi* (CCF 5207)	1.98(1.25–2.5)	6.3(5–10)	1.57(1.25–2.5)	>8
*A. insingulare* (CCF 5417)	6.3(5–10)	0.99(0.625–1.25)	0.79(0.625–1.25)	>8

* MIC values are reported as geometric means of three independent replicates (*n*=3); MIC range concentrations are reported within brackets.

**Table 5:** Antityrosinase activity.

EO	Tyrosinase inhibition (mg KAE/g oil)
EC	31.73 ± 0.63 ^a^
F75	29.41 ± 0.61 ^b^
CS	21.31 ± 1.37 ^c^

Values are reported mean ± SD of three parallel experiments. Different superscripts indicate significant differences in the samples (*p* < 0.05) EO: essential oil; EC: *Eletta campana;* CS: *Carmagnola selezionata*; and F75: *Futura* 75. a,b,c.

**Table 6:** Scavenging/reducing and metal-chelating properties of the tested hemp essential oils [25].

EO	DPPH (mg TE/g EO)	ABTS (mg TE/g EO)	CUPRAC (mg TE/g EO)	FRAP (mg TE/g EO)	Metal Chelating (mg EDTAE/g EO)	Phosphomolybdenum (mmol TE/g EO)
EC	2.53±0.23 ^a*^	32.44 ± 0.03 ^a^	45.51 ± 0.75 ^a^	19.29 ± 0.34 ^a^	11.55 ± 0.84 ^a^	17.95 ± 0.34 ^b^
CS	1.18 ± 0.09 ^c^	32.15 ± 0.08 ^b^	29.91 ± 0.95 ^c^	13.55 ± 0.46 ^c^	7.19 ± 0.42 ^b^	17.52 ± 1.12 ^b^
F75	2.11 ± 0.15 ^b^	32.47 ± 0.04 ^a^	35.05 ± 0.85 ^b^	16.16 ± 0.47 ^b^	10.84 ± 0.46 ^a^	18.80 ± 0.47 ^a^

Values are reported as mean ± SD of three parallel experiments. TE: trolox equivalent; EDTAE: EDTA equivalent. Different superscripts indicate significant differences in the samples (*p* < 0.05). EO: essential oil; EC: *Eletta campana;* CS: *Carmagnola selezionata;* and F75: *Futura 75.* a,b,c. * Values are reported as mean ± SD of three parallel experiments.

**Table 7:** Characteristics of the experimental fields.

EO	GPS Coordinates	Extension (sqm)	Previous Crops (2 Years)	Sowing Scheme (cm Inter/Intra Lines)	Sowing Density
F75	42.363059, 14.093390	4000	Wheat/alfalfa	20 cm/50 cm	15 Kg/ha
CS	42.359222, 14.108528	14,000	Wheat/ Wheat	40 cm/50 cm	20 Kg/ha
EC	42.343824, 14.102808	6000	Not cultivated	20 cm/40 cm	20 Kg/ha

EO: essential oil; EC: Eletta campana; CS: Carmagnola selezionata; and F75: Futura 75.

**Table 8:** Gradient elution of HPLC–UV-MS.

Time (min)	Flow (mL/min)	%A	%B
0	0.750	32.5	67.5
0.5	0.750	32.5	67.5
14	0.750	7	93
22	0.750	7	93
22.1	1.05	32.5	67.5
28	1.05	32.5	67.5
28.1	0.750	32.5	67.5
30	0.750	32.5	67.5

**Table 9:** Mass-to-charge ratios (*m*/*z*) and retention times related to the investigated terpenophenolic compounds.

Standard		*m/z*	Wavelength (nm)	Retention Time (min)
1	CBDA	*357.3*	230	12.4
2	CBGA	343.3–260.1	230	13.6
3	CBG	317.3–234.1–193.1	230	15.4
4	CBD	315.2	230	15.6
5	CBN	311.3–293.25	230	20.0
6	THC-d3	318.9; [315.2: EO pool of THC]	230	20.6
7	CBC	315.3–259.13–193.13	230	21.2
8	THCA	341.3	230	22.5

## Data Availability

The data presented in this study are available on request from the corresponding author.

## Sample Availability

Samples of the compounds are available from the authors.
